# Mechanism of White Matter Injury and Promising Therapeutic Strategies of MSCs After Intracerebral Hemorrhage

**DOI:** 10.3389/fnagi.2021.632054

**Published:** 2021-04-13

**Authors:** Jing Li, Linglong Xiao, Dian He, Yunhao Luo, Haitao Sun

**Affiliations:** ^1^Neurosurgery Center, The National Key Clinical Specialty, The Engineering Technology Research Center of Education Ministry of China on Diagnosis and Treatment of Cerebrovascular Disease, Guangdong Provincial Key Laboratory on Brain Function Repair and Regeneration, The Neurosurgery Institute of Guangdong Province, Zhujiang Hospital, Southern Medical University, Guangzhou, China; ^2^Division of Laboratory Medicine, Clinical Biobank Center, Microbiome Medicine Center, Zhujiang Hospital, Southern Medical University, Guangzhou, China; ^3^Key Laboratory of Mental Health of The Ministry of Education, Guangdong-Hong Kong-Macao Greater Bay Area Center for Brain Science and Brain-Inspired Intelligence, Southern Medical University, Guangzhou, China

**Keywords:** intracerebral hemorrhage, white matter injury, mesenchymal stem cells, corticospinal tract, cell therapy

## Abstract

Intracerebral hemorrhage (ICH) is the most fatal subtype of stroke with high disability and high mortality rates, and there is no effective treatment. The predilection site of ICH is in the area of the basal ganglia and internal capsule (IC), where exist abundant white matter (WM) fiber tracts, such as the corticospinal tract (CST) in the IC. Proximal or distal white matter injury (WMI) caused by intracerebral parenchymal hemorrhage is closely associated with poor prognosis after ICH, especially motor and sensory dysfunction. The pathophysiological mechanisms involved in WMI are quite complex and still far from clear. In recent years, the neuroprotection and repairment capacity of mesenchymal stem cells (MSCs) has been widely investigated after ICH. MSCs exert many unique biological effects, including self-recovery by producing growth factors and cytokines, regenerative repair, immunomodulation, and neuroprotection against oxidative stress, providing a promising cellular therapeutic approach for the treatment of WMI. Taken together, our goal is to discuss the characteristics of WMI following ICH, including the mechanism and potential promising therapeutic targets of MSCs, aiming at providing new clues for future therapeutic strategies.

## Introduction

Intracerebral hemorrhage (ICH) is triggered by the spontaneous rupture of blood vessels, wherein the blood constituents penetrate the brain parenchyma following a path of least resistance and thus destroy both gray and white matter (GM and WM) structures (Qureshi et al., 2009). ICH is the most lethal form of stroke characterized by high morbidity, high disability, and high mortality with no effective treatment. It was demonstrated that more than 77% of ICH patients suffered WM injury (WMI), and a better understanding of WMI and remyelination may shed new light on the treatment of ICH (Smith et al., 2004).

Damage to WM fibers which establish connections among different regions impairs brain connectivity, leading to functional deficits. Dysfunction following cerebral parenchymal hemorrhage derives not only from neuron or synapse losses, but also from primary damage to the WM axons. The area of the basal ganglia where abundant WM fiber tracts cross is the predominant location of hematoma in human ICH. The internal capsule (IC), which lies between the putamen (embraced in the basal ganglia) and the thalamus, contains both descending and ascending fiber bundles conducting sensory, motor, and visual information. Once bleeding occurs in this region, the physiological structure and function of the WM are interrupted to varying degrees, leading to various complications and dysfunctions, such as hemiplegia, hemianopia, hemidysesthesia, and aphasia (Kusano et al., 2009; Balami and Buchan, 2012; Keep et al., 2012; Wu et al., 2017). The corticospinal tracts (CSTs) and corticonuclear tracts’ damage can cause contralateral hemiplegia. Destruction of the optic radiation following deep basal ganglia ICH leads to hemianopia, while hemidysesthesia is caused due to lesions of the central thalamic radiations. It is worth mentioning that IC injury can present all these three clinical manifestations resulting in serious sensorimotor dysfunction in human ICH. WMI is considered the major cause of motor-sensory disorders commonly seen in ICH patients. Therefore, reducing WMI or repairing WM after ICH is critical for reducing long-term neurological deficits, especially for motor function recovery.

In the past few decades, stem cell therapy has been actively explored for the treatment of stroke. Mesenchymal stem cells (MSCs) are pivotal to tissue homeostasis, repair, and regeneration for their self-renewing and multipotent characteristics, which are emerging as the most promising means of allogeneic cell therapy. Basic and clinical research clarify that MSCs are not antigen-presenting cells and would not cause activation of the host’s immune system (Tse et al., 2003). These cells can be harvested and expanded from a variety of adult and perinatal tissues, such as bone marrow, adipose tissue, peripheral blood, fetal tissues, umbilical cord tissues, and placental tissues. The neuroprotection and repairment capacity of MSCs has been widely investigated after ICH. MSCs exert many unique biological effects in tissue replacement, neurotrophy, neurogenesis, angiogenesis, anti-apoptosis, and immunomodulation (Caplan and Dennis, 2006; Mine et al., 2013; Galipeau and Sensebe, 2018; Song et al., 2020), which provides a promising therapeutic strategy in the treatment of WMI. MSCs participate in both innate immunity and adaptive immunity. Their immunomodulatory functions are exerted mainly via the release of bioactive factors and interactions with immune cells, which then change the damaged microenvironment by shifting the balance from toxic to protective regenerative events.

Herein, this review is primarily focused on the pathophysiology and onset mechanism of post-ICH WMI and potential therapy methods of MSCs, in hopes that it can benefit clinical treatment.

## Composition and the Main Function of Brain WM

White matter comprises over 40% of the total volume of adult brain tissue and takes an indispensable part in distributed neural networks that are responsible for neurobehavioral management (Bedell and Narayana, 1998; Herndon et al., 1998). The two main parts- myelinated axon tracts and supporting glial cells including oligodendrocytes (OLs), astrocytes, and microglia- are embraced in WM. The axons (nerve fibers) are surrounded by multiple dense myelin membranes produced by mature OLs (Kang and Yao, 2019), the molecular structure integrity of which makes axons insulated from each other, thus promising the quick and efficient conduction of electrical nerve impulses (transmitting the action potentials) and protecting the nerve fibers from injury (Roncagliolo et al., 2006).

Specifically, three parts constitute WM fiber bundles: projection tracts, commissural tracts, and association tracts (Gerrish et al., 2014). Projection tracts transmit nerve signals from the cortex to other regions of the central nervous system (CNS) (Ge et al., 2002). For instance, CST is the dominant pathway responsible for conveying descending information from the cerebral cortex to the spinal cord. The commissural tracts allow the communication between the left and right cerebral hemispheres (Ge et al., 2002). The association tracts build up connections among cortical lobes within the ipsilateral hemisphere. All these tracts form networks between different regions and serve diverse functions (Ge et al., 2002). Up to now, a large amount of research has clarified the brain functions associated with WM, such as cognitive function, motor function, reading, and practicing abilities (Schmahmann et al., 2008; Carreiras et al., 2009).

## Pathophysiology of WMI After ICH

The pathophysiology change of WMI after ICH is mainly characterized by demyelination, axonal injury, and death of OLs. OLs are the sole source of myelin in the adult CNS, and generally form insulating myelin sheaths marked by myelin basic protein (MBP), enhancing the propagation of action potentials and supporting neuronal and axonal integrity through metabolic coupling under physical circumstances (Bacmeister et al., 2020). OLs contain a high level of iron and are especially sensitive to iron overload, which makes them quite vulnerable to hemorrhagic insults; meanwhile, missed OLs leave axons susceptible to degeneration (Windrem et al., 2020). With the degradation of hemoglobin released from dead erythrocytes, there is a sharp increase in intracellular Fe^2+^ destroying OLs, and iron chelator can inhibit this oxidative toxicity in OLs. Both apoptosis and necrosis are involved in the mechanism of OLs loss. With the onset of ICH, OLs’ expression of caspase-3 in the lesion increases and most of the damaged cells undergo necrosis. Mitochondrial dysfunction is also attributed to OLs apoptosis. Fortunately, OLs maintain the capacity of regeneration and repair after damage to the CNS. OLs death is accompanied by oligodendrocyte progenitor cells (OPCs) proliferation during the acute period in the perihematomal WMI, which is responsible for generating new OLs to remyelinate denuded axons and reconstruct neuronal function.

Dramatical demyelination and axonal damage were first observed at the perihematomal site within three days after ICH, and the axonal damage gradually extended to the adjacent parenchyma over time (Wasserman and Schlichter, 2008). These pathologic changes are highly related to brain edema and neurologic dysfunction, indicating that WMI plays a critical role in neurologic impairment. Hijioka et al. (2016) has investigated the exact relationship between axon pathology and impaired sensorimotor functions; the former mainly referred to axonal transport deficits and structural destruction. The axonal dysfunction in the IC was confirmed to be strongly linked with early motion disturbance after ICH in mice (Hijioka et al., 2016). Remyelination and axonal regeneration are considered valid repair forms in WMI (Joseph et al., 2016). Remyelination is defined as adult OPCs differentiating into new myelin-forming OLs in a regenerative process after CNS demyelination (Gensert and Goldman, 1997; Fancy et al., 2004; Kang and Yao, 2019). To successfully regenerate, damaged axons must reseal denuded stumps, rebuild the cytoskeleton, colligate and transport building substrates, package axon modules, and form growth cones, which is a highly energy-demanding process (Bradke et al., 2012; Lu et al., 2014; He and Jin, 2016). Based on this, Han et al. (2020) raised the “energy deficit” definition and proved that enhancing mitochondrial transport and energetic metabolism can dramatically stimulate axonal regeneration, promoting functional restoration in a spinal cord injury (SCI) animal model. Ultrastructural features of collagenase-induced ICH have been systematically examined in mice over time (Li et al., 2018). Obvious axonal demyelination and degeneration, and the presence of dystrophic neurites in the axons, were demonstrated but OLs proliferation was also observed, which is responsible for the myelination of axons (Li et al., 2018). They first showed a robust inflammatory response of erythrophagocytosis by microglia and macrophages after ICH by transmission electronic microscope (TEM) (Li et al., 2018). Myelination could also be regulated by the growth factor neuregulin (NRG) through binding to the transmembrane tyrosine kinase receptors (ErbB) on unsheathing OLs. Collectively, severe changes of myelin destruction and swelling axons are clear in the ICH animal models, but the exact mechanisms contributing to the loss of motor function are uncertain.

## Pathophysiology Mechanism of WMI After ICH

**FIGURE 1:**
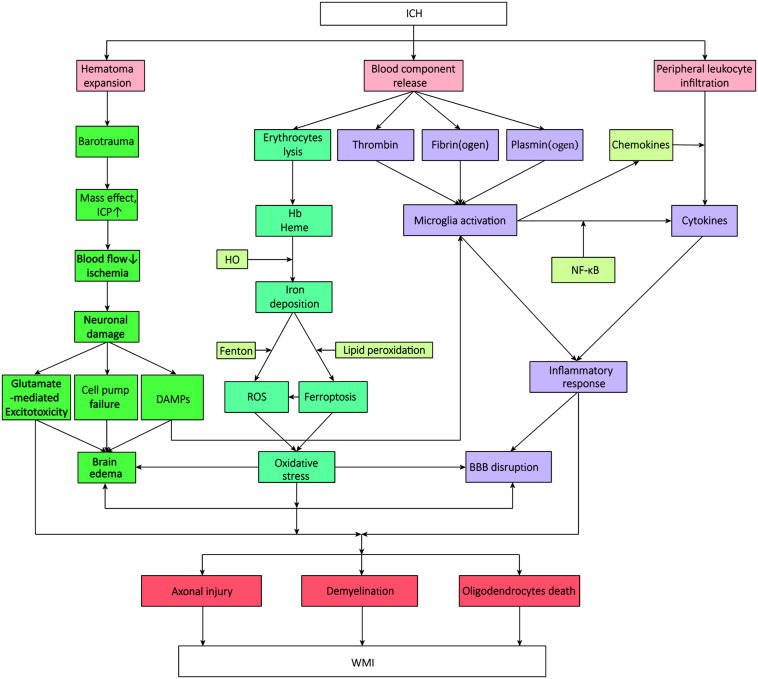
The mechanisms and signal pathways of white matter injury after intracerebral hemorrhage. WMI, white matter injury; ICH, intracerebral hemorrhage; ICP, intracranial pressure; DAMPs, damage-associated molecular pattern molecules; HO, hemeoxygenase; BBB, blood-brain barrier; NF-κB, nuclear factor-κB; ROS, reactive oxygen species.

The influx of blood from vessels into the brain destroys GM and WM. Increasing studies have focused on the implicated pathophysiology of WMI after ICH (Wasserman and Schlichter, 2008; Gerrish et al., 2014; Chen W. et al., 2019; Chen Z. et al., 2019). It is demonstrated that the mass effect and barotrauma during hematoma formation present immediate compression of adjacent brain tissue the moment of ICH onset, which is defined as the primary brain injury. Soon afterward, secondary brain injury- excitotoxicity, oxidative stress, and neuroinflammation- aggravate the WMI leading to neurological deterioration (Aronowski and Zhao, 2011; Babu et al., 2012; Duan et al., 2016; Lan et al., 2017b), accompanied by a succession of pathologic changes which contain hemodynamic changes resulted from ischemia, increased cerebral edema, disruption of the blood–brain barrier (BBB) function, effects of erythrocytes decomposition products, and apoptosis (Aronowski and Zhao, 2011; Keep et al., 2012; Zhou et al., 2014; Chen S. et al., 2015). And it was shown that intracerebral hematoma extended via perivascular spaces and the perineurium (Yin et al., 2013). All these pathologic changes make the nerve fibers within the hematoma lesion distend, distort, and finally disrupt to a point at which they cannot be rescued (Figure 1). Those located in the perihematomal, meanwhile, exert varying degrees of impairment, exactly where researchers continuously focus on and try to intervene.

### Immune Inflammatory Response

Complex immune and inflammatory cascades characterized by the mobilization and activation of inflammatory molecules are triggered at the moment the blood composition is released into the substance, which plays a pivotal role in ICH-induced brain injury (Lan et al., 2017b), especially during the secondary WMI. The primary and secondary damage usually mutually affect the whole developing process. The leakage of BBB after ICH is mainly derived from endothelial cell activation and vascular ONOO^–^ formation, leading to the reduction of key tight junction proteins which generally promise the BBB permeability such as claudin-5, occludin, and zonula occludens (ZO)-1 (Abbott et al., 2010). There is increasing evidence that an inflammatory response can promote the formation of edema by increasing the BBB permeability near the hematoma, thereby aggravating the mass effect, enhancing the process of cell death through secondary ischemia, and further causing inflammatory damage to the surrounding brain tissue (Chu et al., 2014; Wang T. et al., 2015). Many inflammatory cells have been demonstrated to be involved and work in concert to modulate the inflammatory response both in ischemic stroke and ICH. Herein, we introduce several immune cells involved in inflammation in detail as follows.

#### Microglia

Microglia, the resident myeloid phagocytes in the brain parenchyma, constantly and rapidly monitor the brain microenvironment for threats and damage and play a critical role in maintaining homeostasis in the brain by removing pathogens and injured brain tissue debris, and reconstructing the extracellular matrix and synapses, just like a “cop.” When encountered with ICH, they can be activated within minutes and migrate to the lesion together with peripheral macrophages (Lan et al., 2017b), and become highly phagocytic to respond to factors secreted by necrotic neurons or astrocytes, like adenosine triphosphate (ATP), galactin-3, or high mobility group box 1 (HMGB1), which are all embraced in damage-associated molecular pattern molecules (DAMPs, also known as alarmins) (Kong and Le, 2011; Ohnishi et al., 2011; Fang et al., 2013; Zhou et al., 2014; Xiao et al., 2020). Meanwhile, microglia cells secrete multiple cytokines and chemokines associated with the axon-glial injury via the transcription factor nuclear factor-κB (NF-κB), and show enhanced phagocytosis, thereby aggravating structural WMI. DAMPs can initiate the activation of microglia via several toll-like receptors (TLRs) in the neuroinflammatory processes after ICH. The TLR4 on microglia cell surfaces generally interacts with TRIF and MyD88, and then the information is passed through downstream NF-κB, like phosphorylated P65, and several other secreted proinflammatory factors, causing inflammation (Zhou et al., 2014; Lan et al., 2017a). It was proven that traumatic brain injury (TBI) could induce a rapid and persistent up-regulation of Myd88, NF-κB, and proinflammatory cytokines (Ling et al., 2013). Microglial activation can also be induced by thrombin and complement factors via protease-activated receptors (PARs). A newly found adenosine diphosphate (ADP)/ATP-responsive G-protein coupled receptor, P2Y12, helps microglia migrate to the site of injury (Suzuki et al., 2020). Subsequently, microglia cells in an activated state and the toxic molecules secreted further destroy the BBB, leading to peripheral immune cells’ infiltration, thus propagating inflammatory damage.

According to the surface markers and intracellular cytokines expressed, microglia can be polarized into classically activated (M1, pro-inflammatory, and neurotoxic) and alternatively activated (M2, anti-inflammatory, and neuroprotective) phenotypes (Zhang et al., 2017). That the microglia can dynamically and temporally change their phenotypes in response to acute brain injury is exactly the pointcut of brain damage intervention researchers pay attention to. M1 microglia could release high levels of proinflammatory cytokines, that, in turn, hinder axonal regeneration and OLs maturation (Lampron et al., 2015; Chen et al., 2017; Qin et al., 2017). In contrast, polarized M2 microglia typically secrete restorative cytokines and growth factors, remove tissue debris through phagocytosis, and promote remyelination (Olah et al., 2012; Zhao et al., 2015). It was proven that activated microglia act as a double-edged sword and the transfer of M1 to M2 can dramatically alleviate the brain injury caused by inflammation (Zhao et al., 2019).

#### Astrocytes

Astrocytes are recognized as active elements of the brain circuitry, and play key roles in maintaining homeostasis of the extracellular environment, including neurotrophic and structural supporting functions, stabilization of cell–cell communications, and anti-oxidative stress functions (Ransom and Ransom, 2012). Activated astrocytes usually respond to CNS disorders through reactive astrogliosis which represent a series of successive processes including changes of gene and protein expression, proliferation and migration of cells, cellular hypertrophy, and formation of glial scars. Some research demonstrated that reactive astrocytes generate neurotrophic factors (NTFs), isolate injured sites, and prevent harmful inflammation. Whereas it was shown that astrocytes could restrain axonal regeneration and hamper other repair processes in the brain by expressing a wide range of molecules as demonstrated in an earlier study (McKeon et al., 1991). Moreover, axonal regeneration and the reconnection among neurons are further inhibited by the resulting glial scar, inhibiting the formation of which does not always alleviate tissue damage in animal experiments (Wanner et al., 2013; Cregg et al., 2014).

#### T Lymphocytes

Lymphocytes play a vital role in immune surveillance and homeostasis maintenance in the peripheral system, among which T cells mainly participate in adaptive cellular immunity. CD4^+^ T cells dominate the lymphocyte population whether in autoblood- or collagenase-induced ICH models, while CD8^+^ T cells constitute an extremely small infiltrating leukocyte population. Undisputedly, FoxP3^+^CD4^+^ regulatory T cells (Tregs) are the major immunosuppressive lineage of the CD4^+^ T cell compartment. It is generally supposed that T cells rarely enter the brain parenchyma except when pathologic changes occur, and only when the stroke onset, T cells would migrate largely and infiltrate into the lesion site following the activation of microglia (Ito et al., 2019).

Recent research demonstrated that it was the regulatory T cells inside the brain that exerted robust neuronal protection in ischemic stroke by suppressing neurotoxic astrogliosis through producing epidermal growth factor receptor (EGFR) ligand (Ito et al., 2019). This finding suggests that, in the repair of the ICH process, the function of brain T cells should also be highly focused on. Apart from sustaining immune tolerance (Sakaguchi et al., 2010; Lowther and Hafler, 2012), Tregs perform specialized functions in tissue homeostasis and remodeling mainly through restraining the activation and release of cytokines (Liesz et al., 2009; Ito et al., 2019). IL-10 originating from Tregs can trigger hemoglobin-activated microglia/macrophages toward the M2 phenotype. Similarly, it was indicated that Tregs protected against ICH-induced inflammatory injury by modulating microglia/macrophages polarization through the IL-10/GSK3β/PTEN axis which might exert importance in Treg-induced microglia polarization (Zhou et al., 2017). When co-cultured *in vitro*, Tregs also changed the polarization of microglia, decreased the expression of MHC-II, IL-6, and TNF-α, and increased expression of CD206.

CCR5 participates in the regulation of T cells and monocytes/macrophage lines’ migration, mainly expressed on the T cells at rest, monocytes, and immature dendritic cells (Joy et al., 2019). Also, accumulating evidence highlighted a central role for mTOR as a fundamental determinant of cell fate in antigen-activated CD4^+^ T cells. It was shown that suppression of activity in the Akt/mTOR pathway impaired Th17 and Th1 differentiation and promoted the development of Tregs. Fingolimod is recognized as an inhibitor of peripheral immune cell infiltration and can reduce inflammatory injury after ICH (Wei et al., 2011; Fu et al., 2014). These results demonstrated that the number of protective Tregs in the CNS was reduced when the infiltrating inflammatory cells were non-specifically inhibited. It was proven that the transfer of protective Tregs attenuated neurological deficit after ICH.

### Oxidative Stress

Oxidative stress initiated by the blood breakdown component or factors of the plasma plays a central role in the pathogenesis of WMI. Hemolysis after ICH is not that rapid. Rather, the main component of hematoma-erythrocytes starts to lyse 1 day after ICH and continues over days to weeks, followed by the release of decomposition products, including hemoglobin (Hb), heme, and iron, which exert neurotoxic effects on the lesion site and perihematomal regions (Cao et al., 2016). These metabolites, on the one hand, cause consecutive oxidative reactions; on the other hand, they can trigger inflammatory reaction via TLRs. Iron is derived from the collapse of heme by hemeoxygenase (HO)-1 and HO-2. Iron mediates neuronal ferroptosis, generating reactive oxygen species (ROS) and turning Fe^2+^ into Fe^3+^ via the Fenton reaction (Hu et al., 2016). ROS also mediates the inflammatory cascade, giving rise to cell death and perihematomal swelling, which finally leads to WMI.

### Neurotoxicity Mediated by Glutamate

In addition to increased ROS and oxidative stress, the levels of extracellular glutamate can also be augmented after ICH. It was confirmed followed by an increase of oxidative stress leading to sustained neuronal loss that ICH-induced striatal lesion produces distinct changes of EAAT1 and EAAT2 glutamate transporters expression and glutamate uptake activity. Besides, the level of perihematomal glutamate is highly related to the outcome in clinical ICH patients (Wu et al., 2013). It was demonstrated that in hemiplegic patients with basal ganglia bleeding affecting the IC, the earlier mini surgery was conducted, the less glutamate existing in peri-hematoma and the better prognosis, which means the level of glutamate is highly correlated with the outcome of ICH (Wu et al., 2013). Several pieces of research showed blood glutamate grabbing was effective at lessening the excitotoxicity of extracellular glutamate released during ischemic brain injury, although this did seemingly occur in ICH (Castillo et al., 2016; da Silva-Candal et al., 2018). It was indicated that blood glutamate grabbing cannot reduce the hematoma but still can serve as a safe excitotoxic treatment modality following ICH.

## Functions and Possible Mechanisms of Mscs Transplantation Targeting WMI After ICH

Ongoing research efforts are confirming our acknowledgment of the robust potential applications in regenerative medicine of stem cells (Chen et al., 2008), the therapeutic effectiveness of which has been reported in numerous previous experiments conducted in animal models of ICH. Among various stem cells, the multipotency and self-renewal capacity make MSCs a promising candidate for WMI treatment (Ramos-Cabrer et al., 2010; Huang et al., 2013; Mine et al., 2013), which can be expatiated as many unique biological effects including self-recovery by producing growth factors and cytokines, regenerative repair, inherent immunomodulation, and neuroprotection against oxidative stress, and can be further engineered to enhance immunomodulatory functions (Caplan and Dennis, 2006; Lee et al., 2014; Galipeau and Sensebe, 2018; Song et al., 2020), etc.

### Application of MSCs in ICH

There are various sources of MSCs including adult tissues (e.g., bone marrow, adipose tissue, inner organs, and peripheral blood) and neonatal tissues (e.g., umbilical cord, placenta, amniotic fluid, and amniotic membrane). MSCs can be administered either via intracerebral injection into a specific brain region or by intravenous/intraarterial injection (Table 1). In an earlier study on the rat ICH model, intracerebral administration of MSCs enhanced motor coordination and balance, which was attributed to nerve fiber remyelination and axonal regeneration (Liu et al., 2010). Allogeneic and syngeneic BMSCs treatment after stroke in rats improved neurological recovery and enhanced reactive oligodendrocyte and astrocyte-related axonal remodeling with no indication of immunologic sensitization in the adult rat brain (Li et al., 2006). Furthermore, the safety and efficiency of MSCs therapy have also been proven in many clinical trials for stroke, and some of these trials proved the significant neuroprotective effects of MSCs (Table 2). These stem cells are mostly derived from umbilical cord blood, bone marrow, and adipose tissues. Human umbilical cord-derived MSCs (UCMSCs) have been used in clinical trials as a treatment for some neurological diseases since 2011. While the clinical evidence showing the regenerative and immunomodulatory potential of the MSCs on ischemic stroke continues to expand rapidly, the clinical studies on ICH are still scarce. Besides, the proliferation and functions of MSCs are known to decline during the process of senescence. The immunomodulatory functions of MSCs can be compromised due to increased reactive oxygen species and oxidative stress in aged cells. Therefore, early passage MSCs or strategies to prevent senescence must be considered to yield better therapeutic function (Li et al., 2017; Fafián-Labora et al., 2019). And freshly thawed MSCs seem to have an impaired immunomodulatory capacity compared to continuously cultured MSCs (Moll et al., 2016).

**TABLE 1:** An overview of the administration of MSCs involved in animal models.

Diseases model	Model animals	Origin of MSCs	Treatment route	Dose	Functions	Mechanisms	References
ICH (collagenase I)	Wistar rats (270–320 g)	BMSCs, passage 3	Intracerebral injection (right striatum)	5.0 × 10^5^ cells	Improved functional deficits and reduced lesion volume	BMSCs decreased apoptotic cells	Yang et al. (2011)
MCAO	Male Balb/c mice (8 W)	BMSCs, passage 3	Intracerebral injection	2.0 × 10^5^ cells	Promoted regeneration of the infarcted brain	BMSCs triggered endogenous signaling pathways of survival and repair in neurons by secreting soluble neurotrophic factors	Shichinohe et al. (2015)
MCAO	Male C57BL/6J mice (5 and 8 W)	BMSCs, passage 3	Intracerebral injection (peri-infarct site)	1.0 × 10^6^ cells	Improved stroke-associated motor and cognitive dysfunction	EA combined with grafted TrkB-MSCs stimulated the BDNF/NT4-TrkB signaling pathway	Ahn et al. (2019)
ICH (autologous arterial blood)	Adult male SD rats (250–280 g)	BMSCs (femurs of 21-day-old male SD rats), passage 4	Intravenous injection (retro-orbital)	NA	Neuroprotective effects: attenuated neurological deficits and activated axonal regeneration	BMSCs increases of GAP-43 expression through ERK1/2 and PI3K/Akt activation	Cui et al. (2017)
ICH (collagenase IV)	Adult male SD rats (250–350 g)	hPD-MSCs	Intravenous injection (tail vein)	1.0 × 10^6^ cells	Improve neurological recovery; prevent hematoma expansion in the hyperacute stage of ICH and decrease acute mortality	MSCs increased the expression of tight junction proteins associated with the enhancement of cerebrovascular integrity	Choi et al. (2018)
MCAO	Adult male Wistar rats (270–300 g)	HP-BMSCs (femurs and tibias of 2-week-old Wistar rats)	Intravenous injection (tail vein)	1.0 × 10^6^ cells	Promoted locomotion recovery; enhanced angiogenesis and neurogenesis	Hypoxic preconditioning enhanced BMSCs survival and regenerative properties: downregulated inflammatory genes and reduced expression of inflammatory factors, enhanced expression, and release of trophic/growth factors	Wei et al. (2012)
ICH (collagenase IV)	Adult male C57BL/6 mice (8–10 W, 25–28 g)	HP-BMSCs (tibias of post-natal day 21 Wistar rats), passage 5	Intranasal injection	1.0 × 10^6^ cells	Promoted behavioral recovery	HP-BMSCs increased the expression of neurotrophic factors and enhances endogenous neurogenesis	Sun et al. (2015)
ICH (collagenase AOF type A)	Male C57BL/6J mice (7–9 W)	hAD-MSCs, passage 4	Intravenous injection	1.0 × 10^6^ cells	Improved neurological deficits: ameliorates motor and cognitive function	hADSCs suppressed acute inflammation mediated by CD11^+^CD45^+^ subpopulations	Kuramoto et al. (2019)
MCAO	Female SD rats (14–16 W, 225–275 g)	aMSCγ (femurs and tibias of SD rats < 4W)	Intravenous injection (retro-orbital sinus)	5.0 × 10^6^ cells/kg	Minimized the infarct and penumbra; improved functional recovery	aMSCγ reversed the proinflammatory phenotype of microglia and reduce inflammatory signaling; induced OLs differentiation and myelination	Tobin et al. (2020)
MCAO	Male CX3CR1^*eGFP/+*^ CCR2^*RFP/+*^mice (11–13 W, 22–26 g)	IL13-MSCs	Intracerebral injection	5.0 × 10^4^ cells	No obvious differences were observed	IL13-MSCs polarized both microglia and macrophages to a neuroprotective M2 phenotype during the pro-inflammatory status	Hamzei Taj et al. (2018)
ICH (collagenase VII)	Adult male SD rats (270–300 g)	MSCs, passage 10	Intracerebral/intravenous injection (CA/CV/LV)	2.0 × 10^6^ cells	Improved the motor function	MSCs differentiate into neurons, astrocytes and OLs	Zhang et al. (2006)
HI brain injury	C57BL/6 mouse pups (P9)	hMSCs, passage 3	Intranasal injection	1.0/2.0 × 10^6^ cells	Improved sensorimotor function, promoted neuroregeneration, decreased lesion volume, reduced scar formation	hMSCs decreased microglia and astrocytes activity by secreting anti-inflammatory cytokines	Donega et al. (2014)
ICH (collagenase IV)	Adult male C57BL/6 mice (6–8 W, 22–25 g)	BMSCs (femurs and tibias of SD rats), passage 2–5	Intracerebral injection (the ipsilateral lesion area)	2.0 × 10^6^ cells	Attenuated brain water content, reduced hematoma volume, and improved neurological behavior impairment	BMSCs improve the anti-apoptotic ability of reactive astrocytes, trigger GFAP/VIM switch, and inhibit the final formation of the glial scar	Chen et al. (2020)
TBI	Adult male SD rats (220–250 g)	BMSCs (from SD rats), passage 3–8	Intravenous injection (jugular vein)	4.0 × 10^6^ cells	Improved neurological recovery; reduced brain water content	MSCs enhanced expression of TSG-6, and modulate inflammation-associated immune cells and cytokines	Zhang (2013)
ICH (collagenase IV)	Adult male SD rats (250–300 g)	BMSCs (femurs and tibias of 5-week-old SD rats), passage 3	Intravenous injection (jugular vein)	5.0 × 10^6^ cells	Improved neurological deficits; reduced brain water content	TSG-6 produced by MSCs suppressed activation of the NF-κB signaling pathway and the degree of BBB leakage was decreased	Chen M. et al. (2015)
ICH (collagenase)	Adult male SD rats (12 W, 220 g)	HGF transduced hUC-MSCs, passage 4–6	Intracerebral injection	6.0 × 10^5^ cells	Improved motor recovery (motor coordination and balance)	HGF-transduced MSCs enhance nerve fiber remyelination and axonal regeneration	Liu et al. (2010)
ICH (collagenase VII)	Adult male SD rats (190–210 g)	Flk-1^+^ hBMSCs, passage 5	Intracerebral injection (ipsilateral brain parenchyma)	2.0 × 10^5^ cells	Reduced brain edema; improved neurological function	Flk-1^+^ hBMSCs reduced inflammatory infiltration, decreased cell apoptosis, and promoted angiogenesis	Bao et al. (2013)
ICH (collagenase VII)	Male SD rats (230–260 g)	hUC-MSCs	Intracerebral injection	2.0 × 10^5^ cells	Reduced injured lesion; accelerated functional recovery	hUC-MSCs inhibited inflammation and promoted angiogenesis	Liao et al. (2009)
ICH (autologous arterial blood)	Male *Macaca fascicularis* monkeys (4–6 years old, 4.0–4.4 kg)	hBMSCs, passage 6	Intracerebral injection	1.0–5.0 × 10^6^ cells	Improved the recovery from ICH in a primate model	No mention	Feng et al. (2010)
ICH (collagenase IV)	Male SD rats (7 W, 240–280 g)	WJ-MSCs	Intracerebral injection (ipsilateral striatum)	2.0 × 10^5^ cells	Improved behavioral recovery	WJ-MSCs upregulated GDNF and increased differentiation into neuron-like cells	Lee et al. (2015)
ICH (collagenase VII)	Adult male SD rats (250–300 g)	hUCB-MSCs	Intracerebral injection (left lateral ventricle)	5.0 × 10^5^ cells	Improve neurological recovery	hUCB-MSCs modulated the inflammatory environment, promoting neurogenesis and angiogenesis	Kim et al. (2015)
ICH (autologous blood)	Male SHR and WKY rats	BMSCs (femurs and tibias of 8-week-old SHR), passage 3	Intravenous injection (tail vein)	2.0 × 10^7^ cells	Enhanced neurological function recovery	BMSCs improved the integrity of the BBB	Wang C. et al. (2015)
ICH (collagenase VII)	Male SD rats (230–260 g)	hUC-MSCs, passage 3–6	Intravenous/intracerebral injection	2.0 × 10^6^ cells	Improved neurological function and decreased injury volume	hUC-MSCs promoted angiogenesis	Xie et al. (2016)
ICH (collagenase IV)	Female adult Wistar rats (200–250 g)	BMSCs (femurs and tibias of Wistar rats)	Intracerebral injection (lesion zone)	5.0 × 10^6^ cells	Improved neurological function	Platelet-rich plasma-derived scaffolds increased the viability and biologic activity of BMSCs and optimize functional recovery	Vaquero et al. (2013)
subcortical IS with WMI	Male SD rats (200–250 g)	AD-MSCs (adipose tissue of SD rats)	Intravenous injection (tail vein)	2.0 × 10^6^ cells	Improved functional recovery	AD-MSCs reduced cell death, increased cell proliferation, and upregulated levels of white matter-associated markers (NF, MBP, and Olig-2) leading to the restoration of white tract connectivity	Otero-Ortega et al. (2015)
MCAO	Adult male SD rats (270–300 g)	BMSCs (SD rats), passage 3	Intracerebral injection (left lateral ventricle)	3.0 × 10^3^ cells	Alleviated the WMI	BMSCs alleviated neuronal/axonal injury and promote the proliferation of OPCs and formation of the myelin sheath	Yu et al. (2018)
MCAO	Rat pups (P10)	MSCs	Intranasal injection	1.0 × 10^6^ cells	Improved long-term motor functional outcome	MSC enhanced white matter integrity	van Velthoven et al. (2017)
ICH (autologous blood)	Adult male Wistar rats (270–320 g)	hBMSCs	Intraarterial (internal carotid artery)/intravenous (tail vein) injection	1.0 × 10^6^ cells	Improved neurological functional outcome	hBMSCs improved histochemical parameters of neural regeneration and reduced the anatomical and pathological consequences of ICH	Seyfried et al. (2008)
ICH (collagenase VII)	Male ICR mice (7 W, 20–30 g)	hBMSCs, passage 4–11	Intracerebral injection (ipsilateral striatum)	2.0 × 10^5^ cells	Improved motor functional recovery	hBMSCs could be induced to differentiate mostly into neurons and a smaller number of astrocytes *in vitro* and *in vivo* and produce many neuroprotective factors	Nagai et al. (2007)
ICH (collagenase IV)	Adult male Wistar rats (393.1–450.9 g)	hAD-MSCs	Intravenous injection	3.0 × 10^6^ cells	Improved the functional outcome	hAD-MSCs activated the neuronal endogenous progenitor cells	Fatar et al. (2008)
Preterm WMI	Wistar rat pups	hWJ-MSCs	Intranasal injection	1.4 × 10^4^ cells	Improved neurological recovery	hWJ-MSC prevented hypomyelination and microgliosis in a model of WMI in the premature rat brain	Oppliger et al. (2016)

*W, weeks; g, gram; ICH, intracerebral hemorrhage; WMI, white matter injury; MCAO, middle cerebral artery occlusion; IS, ischemic stroke; HI, hypoxic-ischemic; TBI, traumatic brain injury; MSCs, mesenchymal stem cells; BMSCs, bone marrow mesenchymal stem cells; hMSCs, human MSCs; hBMSCs, human BMSCs; HP-BMSCs, hypoxic preconditioned BMSCs; hUC-MSCs, human umbilical cord-derived MSCs; hADSCs, human adipose-derived stem cells; hAD-MSCs, human adipose-derived MSCs; hPD-MSCs, human placenta-derived MSCs; aMSCγ, interferon-γ–activated MSCs; WJ-MSCs, Wharton’s jelly-derived MSCs; hUCB-MSCs, human umbilical cord blood-derived MSCs; Flk-1, fetal liver kinase 1; TrkB, tropomyosin receptor kinase B; EA, electroacupuncture; BDNF, brain-derived neurotrophic factor; NT4, neurotrophin-4/5; GAP-43, growth-associated protein-43; SD, Sprague-Dawley; OLs, oligodendrocytes; IL13, interleukin 13; CA, carotid artery; CV, cervical vein; LV, lateral ventricle; GFAP, glial fibrillary acidic protein; VIM, vimentin; TSG-6, tumor necrosis factor (TNF-α) stimulated gene/protein 6; NF-κB, nuclear factor-κB; BBB, blood–brain barrier; HGF, hepatocyte growth factor; GDNF, glial cell line-derived neurotrophic factor; WKY rat, Wistar-Kyoto rat; SHR, spontaneously hypertensive rat; OPCs, oligodendrocyte progenitor cells.*

**TABLE 2:** An overview of the administration of MSCs involved in clinical trials.

Diseases	Clinical trials	Time from onset	Trial design	Origin of MSCs	Application	Dose	Safety analysis	Efficacy analysis	Functional results	Side effects	Limitation	References
IS	Prospective, randomized, open-label, blinded-endpoint	Acute and chronic phase	Treatment, *n* = 40; control, *n* = 20. Follow-up, 3 months	Autologous MSCs preconditioned with early-phase stroke serum	Intravenous infusion	1.0 × 10^6^ cells/kg	Screening tests monitored vascular occlusion	Multimodal MRI and detailed functional assessments	Safe and feasible	Not mentioned	Short duration of the follow-up evaluation	Kim et al. (2013)
IS	Phase IIa, prospective, randomized, double-blind, placebo-controlled, single-center, pilot	Acute phase (the first 2 weeks)	Treatment, *n* = 10; control (placebo or vehicle), *n* = 10. Follow-up, 2 years	Allogeneic MSCs from adipose tissue	Single intravenous infusion	1.0 × 10^6^ units/kg	AEs, SAEs, neurologic and systemic complications, tumor development	mRS; NIHSS; infarct size; biomarkers	(1) Safe and feasible; (2) significantly improved recovery in the early stages of stroke by repairing ischemic brain tissue	Not mentioned	Small sample size	Díez-Tejedor et al. (2014)
IS	Phase II, prospective, randomized, controlled, observer-blinded	Subacute phase (30–90 days)	Treatment, *n* = 59; control, *n* = 59. Follow-up, 360 days	Allogenic BMSCs	Four intrathecal infusions once a week	1.0 × 10^6^ cells/kg	AEs, Neurological worsening tumor formation or abnormal cell growth, routine tests	NIHSS; mRS; mBI; FMA, ARAT, and MWS; MoCA; infarction volume; tissue metabolism; fiber tract of the injured brain; the level of biomarkers	Safe and feasible	Not mentioned	Not mentioned	Deng et al. (2019)
IS	Phase I/II, multi-center, open-label	Chronic phase (>6 months)	Phase 1, *N* = 15; phase 2, *N* = 21. Follow-up, 1 year	Allogeneic ischemia-tolerant MSCs	Single intravenous infusion	Phase 1: 0.5/1.0/1.5 × 10^6^ cells/kg; phase 2: 1.5 × 10^6^ cells/kg	AEs	NIHSS, BI, Mini-Mental Status Exam, Geriatric Depression Scale scores	(1) Safe and feasible; (2) behavioral gains	Infections, vascular disorders, and pain syndromes, unrelated or unlikely related to the investigational product; urinary tract infection and intravenous site irritation	Uncontrolled design; Mechanism of action was not studied; no appropriate training	Levy et al. (2019)
IVH	Phase I, open-label, single-arm, single-center	23–34 weeks	No control. Premature infants, *N* = 9	Allogeneic hUCB-MSCs	Single intraventricular infusion	3 received 5.0 × 10^6^ cells/kg, 6 received 1.0 × 10^7^ cells/kg	SAEs, DLT, MRI, death after transplantation, anaphylactic shock	Cranial ultrasono graphy, biomarkers	Safe and feasible	Inguinal hernia, late-onset sepsis, and meningitis. These SAEs are not directly related to MSCs	Small sample size; uncontrolled design	Ahn et al. (2018)
Stroke	Phase 1/2a small-scale, open-label, dose-escalation	Chronic phase	No control. Treatment, *N* = 18. Follow-up, 1 year	Modified BMSCs (SB623)	Stereotactic implantation	3 cohorts: 2.5/5.0/10.0 × 10^6^ cells	TEAEs	NIHSS, MRI, FM total score, and FM motor function total score	(1) Safe and well-tolerated; (2) significant improvement in neurological function	All patients experienced at least 1 TEAE. None were related to cell treatment	Small sample size; non-randomized, uncontrolled design	Steinberg et al. (2016)
IS	Prospective, single-center, open-label, blinded-endpoint randomized controlled	Subacute phase, <2 weeks following moderate-severe ischemic carotid stroke	Treatment, *n* = 16; control, *n* = 15. Follow-up, 2 years	Autologous BMSCs	Single intravenous infusion	1.0/3.0 × 10^8^ cells/kg	AEs	NIHSS, mRS, BI, ITT, LMM, motor FM score, task-related fMRI	(1) Safe and feasible; (2) improve motor recovery through sensorimotor neuroplasticity	10 and 16 AEs in treated patients, and 12 and 24 in controls at 6-month and 2-year follow-up, respectively	Use of autologous MSCs; no sample size justification for the primary endpoint; small sample size	Jaillard et al. (2020)
IS	Phase I, open-label, uncontrolled, dose–response, pilot	Acute phase	No control. Treatment, *N* = 6. Follow-up, 1 year	Novel Bone marrOW stem cell (RAINBOW): autologous BMSC product HUNS001–01	Intraparen chymal infusion	3 received 2.0 × 10^7^cells; 3 received 5.0 × 10^7^ cells	AEs	NIHSS, mRS, FIM, BI, FM, MRI, FDG-PET, IMZ-SPECT	Safe and feasible	No AE	Small sample size; uncertain time point of cell administration	Shichinohe et al. (2017)
ICH	Prospective	5–7 days	Treatment, *n* = 60; control, *n* = 40. Follow-up, 6 months	BMSCs	Intracerebral injection (perihemor rhage area in the base ganglia)	Median number of MSCs: 9.47 × 10^5^/L (range, 7.25 × 10^5^ to 1.35 × 10^6^/L), 3.5 mL/patient	Cell viability, re-bleeding or infection, blood pressure control	NIHSS; BI	Reduced neurological impairment and improved activities of daily living	Low grade fever; continuous dull chest pain 4 months after the implantation	Uncertain functional cell types; unclear effectiveness of mononuclear cell therapy in all ICH patients; experimental nature of the stem cell treatment	Li et al. (2013)
IS	Pilot	Acute (<1 week); subacute (1 week to 1 month); stroke sequelae (0.5–2 years)	No control. **Treatment 1:** *N* = 2, 1 dead; **Treatment 2:** *N* = 4. Follow-up, 2 years	Transplantation of NSPCs and UC-MSCs	Intravenous and intrathecal infusions	**Treatment 1:** 0.5 × 10^6^/kg, 4 times; **Treatment 2:** 1 with MSCs (0.5 × 10^6^/kg) + 3 with MSCs (5.0 × 10^6^/ patient) and NSPCs (6.0 × 10^6^/ patient)	Neurological deterioration or infection, tumorigenesis	NIHSS, mRS, BI,	(1) Safe and feasible; (2) improved the neurological functions, disability levels, and daily living abilities	Low-grade fever; minor dizziness	Small sample size; short duration of the follow-up evaluation; uncontrolled design	Qiao et al. (2014)
ICH/IS	Pilot	Within 3 months	No control. Treatment, *N* = 4, 3 with IS, 1 with ICH. Follow-up, 6 months	UC-MSCs	Single intra-artery infusion	2.0 × 10^7^ cells	Angiography, MRI	mRS	(1) Safe and feasible; (2) improve the neurological function of IS patients with the MCA territory infarcts, but not ICH	No obvious AEs	Limited number of enrolled patients; short duration of the follow-up evaluation	Jiang et al. (2013)
IS	Randomized open-labeled, observer-blinded	Acute phase	Treatment, *n* = 16, 4 dead; control, *n* = 36, 21 dead. Follow-up, 5 years	Autologous *ex vivo* cultured MSCs	Intravenous infusion	5.0 × 10^7^ cells	Mortality, SAEs; immediate reaction	mRS, biomarkers, degree of involvement of the subventricular region of the lateral ventricle	(1) Long-term safe and feasible; (2) MSCs may improve recovery after stroke	No obvious AEs	Small sample size; not double-blinded; no exclusion of placebo effects	Lee et al. (2010)
IS	Phase I, unblinded	Subacute or chronic phase	No control. Treatment, *N* = 12, patients with ischemic gray matter, white matter, and mixed lesions. Follow-up, 1 year	Auto serum-expanded autologous human MSCs	Single intravenous infusion	0.6–1.6 × 10^8^ cells	AEs, MRI- tumorigenesis and abnormal cell growth	NIHSS; mRS; MRI; MRA; brain 3D CT angiography	Safe and feasible	Slight itching at the injection site; mild fever and nausea; slight appetite loss; no other obvious adverse events	Unblinded; No overall function or relative functional importance of different types of deficits; No mention of placebo effects or a contribution of recovery of the natural history of stroke	Honmou et al. (2011)
ICH/IS	Pilot	Chronic	No control. Treatment, *N* = 10, 6 with IS, and 4 with ICH. Follow-up, 6 months to 2 years	UC-MSCs, OECs, NPCs, Schwann cell	Intracranial/intravascular infusion	Mixed cells: UC-MSCs, 1.0/2.3 × 10^7^ cells;	Clinic Neurologic Impairment Scale; BI	Clinic Neurologic Impairment Scale; BI	(1) Relatively clinically safe; (2) neurological function amelioration	No AE	Small sample size; methodological limitations; incomplete outcome data; absence of environmental enrichment or cell-only treatment groups	Chen et al. (2013)
Stroke	Unblinded non-randomized experimental controlled	Chronic	Treatment, *n* = 20; control, *n* = 20. Follow-up, 24 weeks	Autologous BMSCs	Intravenous infusion	5.0–6.0 × 10^7^ cells	Routine laboratory tests	Strength, Tone (modified Ashworth), FM, Edinburgh handedness inventory, mBI, functional MRI scanning	(1) Safe and feasible. (2) Stem cells act as “scaffolds” for neural transplantation and may aid in repair mechanisms in stroke	No AE	Small sample size; non-randomized design	Bhasin et al. (2013)
Stroke	Non-randomized experimental controlled	Chronic	Treatment, *n* = 6; control, *n* = 6. Follow-up, 24 weeks	Autologous BMSCs	Intravenous infusion	5.0–6.0 × 10^7^ cells	Routine laboratory tests, tumorigenesis, ectopic tissue formation, behavioral abnormality	FM, mBI, MRC, Ashworth tone grade scale scores, Functional imaging scans	Safe and feasible	No AE	Small sample size; non-randomized design; limitation of dose of cells, site, and mode of transplantation	Bhasin et al. (2011)
TBI	Randomized, single-blind controlled	Sequelae of TBI	Treatment, *n* = 20; control, *n* = 20. Follow-up, 6 months	UC-MSCs	Lumbar puncture infusion	1.0 × 10^7^ cells	FM; FIM	FM; FIM	(1) Safe and feasible; (2) UC-MSCs improved the neurological function and self-care in patients with TBI sequels	Low intracranial pressure reactions (mild dizziness and headache)	Small sample size, a multicenter, and large sample size prospective randomized clinical trial is needed	Wang et al. (2013)

*ICH, intracerebral hemorrhage; IS, ischemic stroke; IVH, intraventricular hemorrhage; TBI, traumatic brain injury; MCA, middle cerebral artery; NSPCs, neural stem/progenitor cells; MSCs, mesenchymal stromal cells; BMSCs, bone marrow MSCs; UC-MSCs, umbilical cord-derived MSCs; hUCB-MSCs, human umbilical cord blood-derived MSCs; OEC, olfactory ensheathing cell; NPC, neural progenitor cell; NIHSS, National Institute of Health Stroke Scale; mRS, modified Rankin Scale; FM, Fugl Meyer assessment; BI, Barthel Index; mBI, modified Barthel Index; FIM, Functional Independence Measure; ITT, intent to treat analyses; LMM, linear mixed models analyses; MRI, magnetic resonance imaging; SPECT, single-photon emission computed tomography; FDG-PET, ^18^F-fluorodeoxyglucose positron emission tomography; IMZSPECT, ^123^I–iomazenil single-photon emission computed tomography; AEs, adverse events; SAEs, serious AEs; TEAEs, treatment-emergent AEs; DLT, dose-limiting toxicity; MRA, magnetic resonance angiography; FIM, functional independence measures.*

### Promoting Self-Recovery and Regenerative Repair

The organism itself possesses a few endogenous mechanisms, such as migration of endogenous stem cells and hematoma clearance, which benefit the repair of injured WM structures. Transplanting exogenous stem cells also exerts significant neuroprotection on ICH although the difficulty in obtainment limits their clinical applications. Other sources of stem cells are required for replacement therapy and MSCs rise in response to the proper time and conditions. MSCs can secrete many trophic molecules when transferred into the body generally by two approaches-orthotopic transplantation and caudal vein transplantation (Smith and Gavins, 2012; Shichinohe et al., 2015)- thus promoting endogenous repair mechanism, which eventually accelerates functional recovery after stroke.

Mesenchymal stem cells transplantation is promising in terms of angiogenesis. Pfeiffer et al. (2019) found human amnion–derived MSCs (hAMSCs), well-known for their favorable angiogenic potential, enhanced human placental endothelial cells (hPEC) viability, and network formation of endothelial cells by paracrine factors *in vitro* (Konig et al., 2015) and promoted angiogenesis *in vivo* (Kinzer et al., 2014; Tuca et al., 2016; Ertl et al., 2018). Brain-derived neurotrophic factor (BDNF), as the most abundant neurotrophin in the CNS, can promote neurogenesis, oligodendrocyte genesis, myelination, and synaptic plasticity through interaction with protein tropomyosin receptor kinase B (TrkB), which is also a receptor of neurotrophin-4 (NT4) (Tolar et al., 2010; Lim et al., 2011). Recently, given the fact that TrkB usually can’t be expressed by the MSCs in an undifferentiated state and the capacity of EA in promoting neurofunctional recovery through specific NTFs, such as VEGF, BDNF, and NT4 (Ahn et al., 2016), investigators combined the electroacupuncture (EA) with genetically modified TrkB gene-transfected MSCs (TrkB-MSCs) in a mouse model of ischemic stroke (Ahn et al., 2019). Consistent with the original assumption, the results showed the combination facilitated further neural survival and differentiation via stimulating the BDNF/NT4-TrkB signaling pathway rather than simply the administration of MSCs (Ahn et al., 2019). EA could directly stimulate the proliferation and differentiation of endogenous neural NSC in a rat model of ischemic stroke (Tan et al., 2018). BDNF can also exert neurotrophic effects on neuronal survival and neurite outgrowth, particularly the CST axons (Gupta et al., 2009). This procedure can be mediated by growth-associated protein-43 (GAP-43) which is highly distributed in the presynaptic membrane associated with neurite extension and long-term synaptic enhancement (Ramakers et al., 2000; Gupta et al., 2009; Morita and Miyata, 2013). Cui et al. (2017) elaborated that MSC graft increased GAP-43 expression via the ERK1/2 and pro-survival phosphatidylinositol 3-kinase (PI3K)/Akt signaling pathways after transplanting BMSC into a rat model of autologous blood injection. It means that MSC transplantation alleviated axonal damage and enhanced synaptic plasticity to some extent. GAP-43 may be considered a potential therapeutic target for MSCs in the treatment of axonal injury following ICH.

The CST is the only descending conduction pathway, in which some axons directly take shape synapses with spinal motoneurons, evolved in the major system for skilled voluntary movement in human and motor functions in rodents. Motor deficits in a stroke critically lie in an interruption of CST integrity, i.e., the motor fibers descending from the cortex to the spinal cord (Hijioka et al., 2016; Jang et al., 2018; Chen W. et al., 2019). Our previous research detected WM degeneration which lasted for at least 5 weeks after ICH, i.e., even during the chronic phase following stroke (Ng et al., 2020). And the longitudinal pathological alternations of the CST in the cervical portion of the spinal cord after unilateral striatal hemorrhage in adult mice were first illustrated, implying that the structural integrity of the CST was compromised extensively after ICH (Ng et al., 2020). In general, the establishment of compensatory re-innervation in the bilateral hemispheres after brain injury is mainly achieved through axonal sprouting of surviving neurons, new synapse formation, and factors produced by the brain. MSCs have been reported to promote neurogenesis and to alleviate side effects in injured brain regions, where both differentiation and secretion of MSCs involve axonal plasticity. In addition to the neuroprotective and neurotrophic effects, Choi et al. (2018) proved that MSCs may also prevent hematoma expansion in the hyperacute stage of ICH by enhancing endothelial integrity of cerebral vasculature by uplifting the expression of tight junction proteins (ZO-1, occludin).

### Immunomodulating Properties of MSCs

The scientific fact that neuroinflammation makes a principal contribution to the progress of ICH-induced brain damage is well acknowledged. Thus, modulating the immune response could help improve brain injury outcomes following ICH. The highly anti-inflammatory and immunomodulatory properties make MSCs suitable therapeutic candidates in inflammatory diseases, through regulating infiltration of microglia and neutrophils and increasing anti-inflammatory cytokines levels, while also downregulating the expression of proinflammatory cytokines.

Emerging knowledge in targeting neuroinflammation argues MSCs are effective modifiers of microglial phenotype by maintaining a resting, pro-regenerative microglial phenotype, or by controlling the microglial activation following stroke (Wei et al., 2012; Yan et al., 2013). Neurological deficits of collagenase-induced ICH-bearing mice during the subacute phase were improved by human adipose-derived stem cells (hADSCs) which suppressed the acute inflammation mediated by CD11^+^CD45^+^ cells subpopulations (Kuramoto et al., 2019). In a middle cerebral artery occlusion (MCAO) model of rats, microglia activation and inflammatory signaling were dramatically reduced by transplanted MSCs activated by interferon (INF)-γ, along with oligodendrogenesis and the minimization of the infarct and penumbra (Tobin et al., 2020). The activation of microglia is largely determined by CX3CR1, and MSCs are known to shift activated inflammatory M1 macrophages to an M2 macrophage-like phenotype through prostaglandin E2 (PGE2). In a global cerebral ischemia (GCI) mice model conducted by Du et al. (2020), CX3CR1 downregulation markedly reduced activation of microglia and inflammatory responses and promoted the generation of mature OLs from OPCs, and thus protected myelin from ischemia-induced damage. Similarly, Hamzei Taj et al. (2018) transplanted the BMSCs line which was genetically engineered to express the anti-inflammatory cytokine IL-13 (further named as IL13-MSCs) to CX3CR1^*eGFP/+*^ CCR2^*RFP/+*^ knock-in fluorescent protein reporter mice to distinguish brain-resident microglia from infiltrated macrophages after ischemic stroke. They found the transplantation of IL13-MSCs shifted microglia and macrophages toward an anti-inflammatory, neuroprotective phenotype at 14 days after ischemia (Hamzei Taj et al., 2018). Compared with MSCs, IL13-MSCs were proven to better limit oligodendrocyte loss and demyelination in a model for neuroinflammation and demyelination of cuprizone-treated mice, and promote histopathological and functional recovery in SCI of mice (Hamzei Taj et al., 2018). Engineered MSCs were also applied in malignant glioma tumor models (Sun et al., 2011).

As one of the main components of glial cells, astrocytes’ regulation is also a promising target in WMI treatment (Zhang et al., 2006), although there is not adequate research recorded. Donega et al. (2014) reported that intranasal administration of human-MSC successfully reduced the expression of GFAP (a biomarker of astrocytes) and the formation of glial scars. Interestingly, in experiments conducted by Chen et al. (2020), transplanting BMSCs led to an elevation of GFAP level of expression; this difference might lie in the inherent double-edged features of activated astrocytes. Fortunately, the transplantation of MSCs into the CNS of ICH mice significantly improved cognitive and motor function and decreased hemorrhagic volume, which is consistent with previous research (Bedini et al., 2018). The key challenge in the treatment of ICH is to therefore understand how to magnify the advantages and minify the disadvantage of reactive astrocytes. Another factor that presents a double-edged sword function due to producing both pro-inflammatory and anti-inflammatory cytokines is IL-33; it was proven that IL-33 improved wound healing through enhanced M2 macrophage polarization in diabetic mice (He et al., 2017). It is a member of the IL-1 family mainly expressed in astrocytes, microglia, and OLs in CNS (Schmitz et al., 2005). Besides, a late research voted for IL-33 as a neuroprotective target which shifted microglia polarization from M1 to M2 and thus promoted OLs differentiation and WM repair banding with its ligand ST2 after ICH (Chen Z. et al., 2019).

The immunomodulatory functions of MSCs can also be exerted by secreting multifunctional paracrine signaling factors (Zhang, 2013; Zhao et al., 2013; Liu et al., 2014; Zhou et al., 2019), including cytokines, growth factors, and chemokines, which combine to regulate the immune cells’ function. Systemically delivered MSCs can pass through the BBB while very few of these cells are detected homing to and survive in the lesion site of the brain (Chen et al., 2001). Functional activities are still improved by transplantation therapy. It has been commonly accepted that the functional benefits of MSCs’ transplantation are due to increased trophic support from these cells that reduce overall inflammation, thereby eliminating the potentially toxic environment (Caplan and Dennis, 2006; Hess and Borlongan, 2008). Some studies described the bystander mechanism of MSCs that MSCs is related to some soluble factors such as IL-10, indoleamine 2,3-dioxygenase (IDO), PGE2, transforming growth factor-β1 (TGF-β1), tumor necrosis factor-α (TNF-α), and TNF-α stimulated gene/protein 6 (TSG-6) (Nemeth et al., 2009). These molecules are encapsulated in cell-secreted extracellular vesicles (EVs), which are usually divided into exosomes, microvesicles (MVs), and apoptotic bodies according to the size and cell of origin. TSG-6 is an anti-inflammatory factor that can suppress neutrophil migration into the inflammation region, interact through the CD44 receptor on resident macrophages, and inhibit the NF-κB signaling pathway (Chen M. et al., 2015). The BMSCs, injected from the jugular vein of ICH-bearing rats, attenuated the inflammatory response and decreased BBB disruption by secreting TSG-6 after being trapped in the lung (Chen M. et al., 2015). Most recently, researchers have verified the MSC-derived EVs (MSC-EVs), which mediate cell-to-cell inflammation and trophic signaling, to be feasible therapeutic targets for functional recovery after cortical injury in a monkey model (Medalla et al., 2020). Besides, MSC-EVs can also trigger macrophage polarization by increasing the formation of anti-inflammatory M2 phenotype over M1-like pro-inflammatory phenotype via downregulation of level of IL-23 and IL-22.

An *in vitro* experiment was performed which preclinically investigated the potential effect of MSCs on Treg and showed that MSCs induced the generation of Treg via epigenetic conversion of human conventional CD4 T cells, possibly partly through TGF-β and/or PD-1/PD-L1 pathway (Azevedo et al., 2020). PD-L1 was found to result in generating more Th2 and Treg cells but fewer Th1 and Th17 cells from naive CD4^+^ T cells via inhibiting the mTOR pathway *in vitro* experiment. Administrating PD-L1 could promote the development of Treg cells and inhibit the differentiation of Th17 cells and thus significantly alleviate symptoms and suppress disease progression in several murine models (Fujiwara et al., 2014; Ding et al., 2016; Han et al., 2017). Besides, PD-L1 usually promotes the phosphorylation of immune-receptor tyrosine and conveys negative modulation signals, leading to cell inactivation via STAT or Janus kinase (JAK). When the STAT1 phosphorylation was hampered, overexpressing PD-1/PD-L1 could decrease the M1 microglia, indicating a potential approach of transferring to the anti-inflammatory phenotype via inhibiting STAT1. The findings mentioned above may attract researchers to conduct further investigation on the specific mechanism and therapeutic effect of MSCs transplantation based on PD-1/PD-L1 after ICH.

### Reducing Oxidative Stress

The antioxidative stress properties of MSCs have been validated in many previous studies (Shalaby et al., 2014), as diminishing ROS via the Nrf2 signaling pathway and protecting the body from oxidative stress was proven in many diseases such as acute lung injury (ALI), acute respiratory distress syndrome (ARDS), acute myocardial infarction (AMI), and acute liver failure (ALF). Various preconditioning strategies have been used to enhance the therapeutic efficacy of MSCs. Hypoxia preconditioning is thought to enhance MSCs’ survival and the expression of various trophic factors of MSCs, and even to strengthen the engraftment and paracrine properties (Hu and Li, 2018).

## Conclusion and Perspectives

For the past two decades, great progress in understanding the mechanisms of ICH-induced brain injury has been made (Balami and Buchan, 2012; Fang et al., 2013; Chen S. et al., 2015; Duan et al., 2016; Bobinger et al., 2018). But not until recently has the importance of WM damage in ICH, which exerts a high correlation with functional outcomes, been acknowledged. The WM is involved in the transmission of motor and sensory information between the cerebral cortex and spinal cord. Therefore, whether in hemorrhagic or ischemic stroke, WMI can cause serious cognitive dysfunction, emotional disorders, and motor disturbance. Without the parallel protection of WM, true lasting neurorestoration cannot be achieved.

Mesenchymal stem cells have proven to be an extremely promising therapy for WMI due to their multipotency and self-renewal capacity. Furthermore, they exert reduced immunogenicity because of a low MHC class I expression and the absence of MHC class II molecules and co-stimulatory factors. Most importantly, MSCs can produce many immunomodulatory, neurotrophic, and angiogenic factors and have a potential immunomodulatory effect on immune cells (Caplan and Dennis, 2006; Chen et al., 2008; Bedini et al., 2018; Galipeau and Sensebe, 2018). And the neuroprotective effects have been well recognized in numerous pieces of research. To enhance the therapeutic effects of MSC transplantation by boosting the immunomodulatory properties of MSCs, investigators can also make some improvements to the MSC and the results show enhanced therapeutic effect, especially for the inflammatory modulation.

It is true that numerous signaling molecular pathways are involved in inflammatory responses and further exacerbate secondary brain damage (Zhou et al., 2014; Zhu et al., 2019). Although the modulation of immunological response after ICH showed promising results in a small proof-of-concept study, larger trials need to be done to further verify this. There are still several questions that remain to be addressed. First, studies in WMI are insufficient, whether effective drug targets of MSCs in the diseases mentioned above are equally effective in WM damage after ICH requires further verification. Second, human ICH pathomechanisms cannot be entirely mimicked by experimental models. The proportion of WM in rodent animals, especially in rats and mice, is much smaller than that of humans. It is essential to apply animals whose brain structures fit better with humans in future studies. Third, a majority of studies on post-ICH WMI after ICH concentrate on single−factor intervention; agents with multiple targets or combined drug therapy strategies remain to be designed and tested in further research. Last but not least, cell resources, invasive extraction procedures, and cell quantity make future research of this therapy challenging. An improvement on MSCs calls for further investigation so that it can be better applied in ICH treatment.

## Author Contributions

The work presented here was carried out in collaboration with all authors. HS conceived and designed the review. JL wrote the manuscript. LX, DH, and YL helped with literature searching and summarizing. All authors read, commented on, and approved this manuscript.

## Conflict of Interest

The authors declare that the research was conducted in the absence of any commercial or financial relationships that could be construed as a potential conflict of interest.
